# A Wearable Capacitive Sensor Based on Ring/Disk‐Shaped Electrode and Porous Dielectric for Noncontact Healthcare Monitoring

**DOI:** 10.1002/gch2.201900079

**Published:** 2020-03-18

**Authors:** Ya‐Nan Zheng, Zhe Yu, Guoyong Mao, Yunyao Li, Dhanapal Pravarthana, Waqas Asghar, Yiwei Liu, Shaoxing Qu, Jie Shang, Run‐Wei Li

**Affiliations:** ^1^ CAS Key Laboratory of Magnetic Materials and Devices Ningbo Institute of Materials Technology and Engineering Chinese Academy of Sciences Ningbo 315201 P. R. China; ^2^ College of Materials Science and Opto‐Electronic Technology University of Chinese Academy of Sciences Beijing 100049 P. R. China; ^3^ Zhejiang Province Key Laboratory of Magnetic Materials and Application Technology Ningbo Institute of Materials Technology and Engineering Chinese Academy of Sciences Ningbo 315201 P. R. China; ^4^ State Key Laboratory of Fluid Power and Mechatronic System Key Laboratory of Soft Machines and Smart Devices of Zhejiang Province Department of Engineering Mechanics Zhejiang University Hangzhou 310027 China; ^5^ College of Information Engineering Nanjing University of Finance and Economics Nanjing 210046 China

**Keywords:** capacitively coupled effect, dielectric materials wearable sensors, noncontact healthcare monitoring

## Abstract

Wearable sensors are gradually enabling decentralized healthcare systems. However, these sensors need to be closely attached to skin, which is unsuitable for long‐term dynamic health monitoring of the patients, such as infants or persons with burn injuries. Here, a wearable capacitive sensor based on the capacitively coupled effect for healthcare monitoring in noncontact mode is reported. It consists of a ring‐shaped top electrode, a disk‐shaped bottom electrode, and a porous dielectric layer with low permittivity. This unique design enhanced the capacitively coupled effect of the sensor, which enables a high noncontact detectivity of capacitance change. When an object approaches the sensor, its capacitance change (Δ*C*/*C*_i_ = −38.7%) is 3–5 times higher than that of previously reported sensors. Meanwhile, the sensor is insensitive to the stretching strain and pressure (Δ*C*/*C*_i_ < 5%) due to the unique ring‐shaped electrode and the incompressible closed cells of the porous dielectric material, respectively. Finally, various human physiological signals (pulse and respiratory) are recorded in noncontact mode, where a person wears loose and soft clothes implanted with the sensor. Thus, it is promising to build smart healthcare clothes based on it to develop wearable decentralized healthcare systems.

## Graphical abstract

A wearable capacitive sensor for noncontact healthcare monitoring consists of a ring‐shaped top electrode, a disk‐shaped bottom electrode, and a porous dielectric layer to enhance the capacitively coupled effect. When an object approaches, its capacitance change is 3–5 times higher than that of reported capacitive sensors. Smart clothing implanted with this sensor successfully records the physiological signals by noncontact mode.

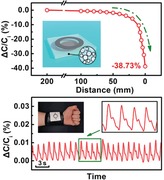

## 1 Introduction

Research and development of wearable sensors are gradually revolutionizing the methods of healthcare monitoring due to their comfortable, portable, timely, and high‐performance characteristic features.^[^ ^1^, ^2^, ^3^, ^4^, ^5^ ^]^ Compared to the traditional centralized healthcare monitoring systems, the decentralized healthcare monitoring systems can be realized based on wearable sensors, which can provide comfortable and personalized medical services at anytime and anywhere. In addition, they can detect the abnormal physiological signals in the early stage of disease for the better treatment of patients.^[^ ^1^, ^2^, ^3^, ^6^, ^7^, ^8^ ^]^ Existing wearable healthcare sensors accomplish the monitor via perceiving the microscopic deformation of epidermis, which is caused by breathing, pulsing, etc.^[^ ^8^, ^9^, ^10^, ^11^ ^]^ Due to these physiological signals of human are very weak, the wearable sensor usually needs to be closely attached to skin.^[^ ^9^, ^10^, ^11^, ^12^, ^13^, ^14^ ^]^

However, attaching the sensors for a too long time may lead to an itch and even an inflammation on human skin due to poor gas/water permeability.^[^ ^15^, ^16^ ^]^ Importantly, human movements can also interfere with the performance of sensor in terms of detecting the weak signals.^[^ ^17^, ^18^ ^]^ To address this, researchers have chosen new materials for constructing the wearable sensors to improve the gas/water permeability or prepare stretchable patterns to avoid interference.^[^ ^16^, ^18^ ^]^ For example, Jung et al. utilized a porous material to print a wearable sensor with S‐shaped pattern.^[^ ^18^ ^]^ Their sensor exhibited a good gas/water permeability and strain‐insensitivity. The noncontact mode, in our opinion, is the most effective method to detect physiological signals, which is also appropriate for the patients, such as infants or persons with burn injuries.^[^ ^1^ ^]^ When a person wears such a wearable sensor implanted in loose and comfortable clothing, it ensures the sensors do not touch human skin and enables the protection of skin. Meanwhile, the basic physiological signals can be accurately detected in real time during exercise. Therefore noncontact detection of physiological signals is considered the key feature in the development of upcoming wearable decentralized healthcare monitoring systems.

In this paper, we report a wearable capacitive sensor to detect physiological signals by noncontact mode. Our sensor is prepared by stencil printing technology and consists of a ring‐shaped top electrode, a disk‐shaped bottom electrode, and a porous dielectric layer. The engineered wearable sensor enables enhancement of the capacitively coupled effect of the sensor to achieve noncontact health monitoring. This enhancement is attained by utilizing a unique shaped electrode and selecting a dielectric material with low relative permittivity (ε_r_), which have been verified by numerical simulations of COMSOL. The obtained sensor exhibits capacitance change as high as 38.7% when the object approaches the sensor in noncontact mode, which is 2–5 times higher than that of reported capacitive sensors. Furthermore, our sensor is insensitive to stretching strain (Δ*C*/*C*_i_ = 4.55% @ 10% strain) and pressure (Δ*C*/*C*_i_ = 3.87% @ 20 kPa) to ensure the stable performance even after repeated stretching (10^3^ times) and pressure (10^3^ times). Finally, our wearable sensors are successfully demonstrated for noncontact healthcare monitoring of respiratory and pulse signals, when the prepared sensor is placed on the surface of loose coat and soft wristband, respectively. In addition to healthcare monitoring, we think our capacitive sensor with the ability of noncontact signal detection has a huge potential to be applied in the field of soft robots.

## 2 Results and Discussion

Generally, noncontact healthcare monitoring devices utilize thermal infrared imaging, doppler radar detecting, and capacitively coupled sensing mechanisms to detect the physiological signals.^[^ ^19^, ^20^, ^21^ ^]^ Among them, the capacitively coupled sensing mechanism is an ideal method to develop a wearable noncontact healthcare sensor because of its simple structure, lightweight, and low power consumption.^[^ ^21^, ^22^, ^23^ ^]^ When such sensor based on the capacitively coupled effect detect the pulsing or breathing signals in noncontact mode, it will cause the undulation of the epidermis to change the distance between skin and capacitively coupled sensor. This leads to a change in the capacitance of the sensor, which responses regularly with the change of the human physiological signals to achieve noncontact health monitoring. The capacitively coupled sensing can be categorized as self‐capacitance and mutual capacitance (Figure S1, Supporting Information).^[^ ^24^ ^]^

The self‐capacitance sensor consists of a single electrode and its capacitance value is relative to the ground, as shown in Figure S1a of the Supporting Information. This capacitance value increases when a human skin approach toward it.^[^ ^22^, ^24^ ^]^ In contrast, the mutual capacitance sensor consists of a pair of electrodes, where capacitance is measured between the electrodes.^[^ ^24^, ^25^ ^]^ Its capacitance value decreases when a human skin approach toward it (Figure S1b, Supporting Information). This arises because skin disturbs its fringing electric field and charge transfer occurs between the two electrodes.^[^ ^24^, ^25^, ^26^, ^27^ ^]^ Compared to the self‐capacitance sensors, the mutual capacitance sensors are less susceptible to the common‐mode of noise environment and also from the change of air circulations.^[^ ^20^, ^28^ ^]^ However, its detectivity is weak,^[^ ^29^ ^]^ for example, the signal change of mutual capacitance sensors, i.e., capacitive sensors, is lower than 15% when a finger approaches it from a distance of 10 cm.^[^ ^27^, ^30^, ^31^, ^32^ ^]^ Therefore, it is important to improve the noncontact detectivity of the capacitive sensor for noncontact healthcare monitoring through a wearable sensor.

**Figure 1:**
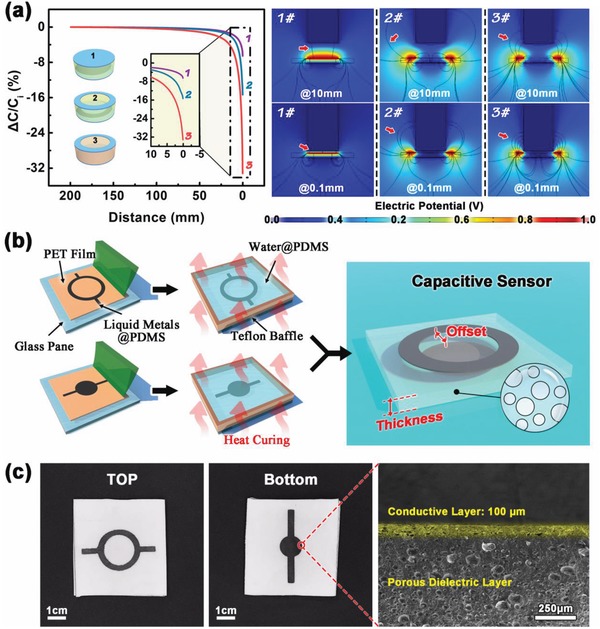
a) Simulated results of three capacitive sensor models. The picture on the left shows the variation of relative capacitance change rate with the distance from an object to the sensor. The pictures on the right show the distributions of the fringing electric field when the object is at different distances. b) Schematic illustration of the fabrication procedure for preparing the wearable capacitive sensor. c) Optical photos and cross‐sectional SEM images of the prepared wearable capacitive sensor.

The sensitivity of the capacitive sensor can be enhanced by changing the shape of electrodes and the relative permittivity of a dielectric material, because these two parameters regulate the distribution of fringing electric field and the couple binding capacity of two electrodes, respectively.^[^ ^24^, ^25^, ^26^ ^]^ To verify this, we have simulated the capacitance changes of three capacitive sensor models in COMSOL, when these sensors are approached by an object. The calculation results are shown in **Figure** **1**a. The first capacitive sensor consists of a disk‐shaped top electrode, a disk‐shaped bottom electrode, and a dielectric layer with high ε_r_ of 3.0. The second capacitive sensor consists of a ring‐shaped top electrode, a disk‐shaped bottom electrode and a dielectric layer with the same high *ε_r_* of 3.0; The third capacitive sensor consists of a ring‐shaped top electrode, a disk‐shaped bottom electrode and a dielectric layer with low ε_r_ of 2.0. When an object approaches these sensor models, the capacitance values of these sensors decrease by 6.55%, 15.34%, and 33.17%, respectively. These differences can be better seen from their projected electric fields when the object is at different distances (the right of Figure 1a). When the object approaches the different sensor models, the electric field lines around the object increase and become denser with the further decrease of distance, as shown by the red arrows marked in the pictures. At the same distance, the electric field lines around the object of the first sensor model are the lightest, and that of the third sensor model is the strongest. This means the strength of the capacitively coupled effect enhanced between the object and the sensor with approaching toward the sensor, and the enhancement is most significant in the third sensor model. Simulation results confirm that the third sensor model is more feasible to develop a wearable capacitive sensor for healthcare monitoring.

The sensor is prepared by stencil printing technology, which is a highly efficient and low‐cost technique. The preparation process is shown in Figure 1b, and the detailed manufacturing steps are discussed in the Experimental Section; and Figure S2 of the Supporting Information. The electrode material of the sensor consist of a printable elastic conductor, which is a composite of polydimethylsiloxane (PDMS) filled liquid metals (LMs@PDMS). The detailed property and synthesis of this elastic conductor were previously reported by our team.^[^ ^33^, ^34^ ^]^ Before the heat curing of electrode material, its rheological and viscosity properties should meet the requirements of conductive inks for printing technology (Figure S3, Supporting Information). This elastic composite conductor exhibits excellent dynamic stability. As shown in Figure S4 of the Supporting Information, its resistance changes are lesser than 1% while stretching or pressing, which provides a necessary guarantee of stability for the sensor during deformation. The dielectric material PDMS is porous with low relative permittivity, which is prepared by heat curing the mixture of PDMS and water. Water vaporizes at high temperature and creates pores in the PDMS matrix. Figure 1c shows the prepared capacitive sensor, which has a conductive electrode layer of 100 µm and a porous dielectric layer.

**Figure 2:**
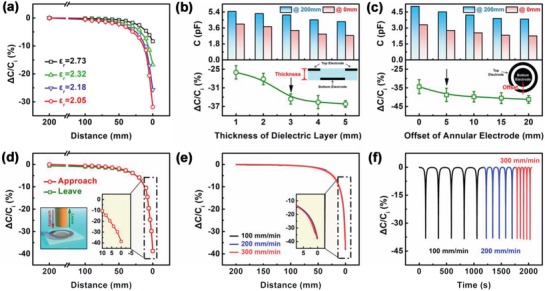
a) Relative capacitance change rate of sensors based on different PDMS dielectric materials as a function of the distance from an object to the sensor. b) Capacitance of sensors with different thicknesses at distances of 200 and 0 mm (upper), and relative capacitance change rate of the sensor as a function of the thickness of dielectric layer (below). c) Capacitance of sensors with different offsets at distances of 200 and 0 mm (upper), and relative capacitance change rate of the sensor as a function of the offset between two electrodes (below). d) The relative capacitive change rate of the sensor with optimal parameters as a function of the distance from an object to the sensor during an approach‐leave operation. e) Relative capacitive change rate during approach‐leave operations with different velocities. f) Relative capacitive change rate during repeated approach‐leave operations with different velocities.

There are three factors that play a significant impact on the device performance of our sensor, which includes the relative permittivity of porous PDMS, the thickness of the sensor, and the horizontal offset between the two electrodes. First, the effect of the relative permittivity of porous PDMS on the sensitivity of the capacitive sensor is investigated. The relative permittivity of PDMS can be controlled by the preparation process because it is closely related to the mixing volume ratio of water in the water@PDMS mixture. In Figure S5 of the Supporting Information, the cross‐sectional view of scanning electron microscope (SEM) images for different mixing volume ratios of water from 0 vol%, 20 vol%, 40 vol%, and 60 vol% are shown. Further, the values of transmittances and the relative permittivity of porous PDMS based on water@PDMS mixture are decreased with the increase in the different mixing ratios of water. When mixing volume ratio of water is increased (0 vol%, 20 vol%, 40 vol%, and 60 vol%), the pores of porous PDMS increase significantly (Figure S5a, Supporting Information), which causes the decrease of transmittance and relative permittivity. Their transmittances are 93.77%, 44.93%, 26.82%, and 8.92%, respectively (Figure S5b, Supporting Information). Their relative permittivities are 2.73, 2.32, 2.18, and 2.05, respectively (Figure S5c, Supporting Information). Therefore, the effect of relative permittivity on the performance of different capacitive sensors are investigated, which used water@PDMS mixture with different mixing ratios of water (0 vol%, 20 vol%, 40 vol%, and 60 vol%) as porous dielectric layers. The results are shown in **Figure** **2**a. It can be found that the noncontact detectivity of the capacitive sensor increases with the increase in the mixing volume ratio of water. The maximum capacitance change (Δ*C*/*C*_i_ = −31.86%) occurs in the sensor made up of porous PDMS prepared by heat curing with 60 vol% water@PDMS mixture (ε_r_ = 2.05). But uncured 60 vol% water@PDMS mixture is easy to occur phases separation of water and PDMS, which makes it incompatible with the preparation process (Figure S6, Supporting Information). The mixing ratio of water is selected as 40 vol% for the porous PDMS dielectric layer, which has the relative permittivity of 2.18. The sensor based on this exhibits a high sensitivity (Δ*C*/*C*_i_ = −25.67%) when an object approaches it from a distance of 200 mm.

Further, the effect of the sensor thickness and the offset between the two electrodes on the sensitivity of the capacitive sensor is investigated. When the object is at a 200 and 0 mm distances from the sensors with different thickness or offsets, their capacitance values were measured, and from that the relative capacitance change rates (Δ*C*/*C*_i_) were determined as shown in Figure 2b,c, respectively. From that, it can be seen that the capacitance value decreases and the relative capacitance change rate increases with the increase in the thickness of sensors (1, 2, 3, 4, and 5 mm) or with the increase in the offset between the top and the bottom electrodes (0, 5, 10, 15, and 20 mm). When the thickness is larger than 3 mm, the capacitance change tends to be little. Meanwhile, when the offset is greater than 5 mm, the change rate also tends to be little. Therefore, the optimal thickness and the offset of capacitive sensors are 3 and 5 mm, respectively.

Figure 2d displays the excellent noncontact detectivity of our capacitive sensor made with optimal parameters, such as the water mixing ratio of 40 vol%, the thickness of 3 mm, and the offset of 5 mm. When an object approaches to the sensor, its capacitance change rate becomes as high as 38.7%. At the same time, the capacitance of the sensor will recover to its initial value when the object leaves to the distance of 200 mm. Whether the object is approaching slowly or rapidly for repeated times (100, 200, and 300 mm min^−1^), the noncontact detectivity of the sensor does not change significantly, as shown in Figure 2e,f. Moreover, the effects of temperature and humidity on the performance of the sensor were investigated. As it is obvious from Figure S7a of the Supporting Information, the temperature has little effect on the sensor (Δ*C*/*C*_i_ = 37.04% ± 0.12%). In comparison significantly (Figure S7b, Supporting Information), the noncontact detectivity of the capacitive sensor decreases after soaking in water for 1 d, but there is no significant change (Δ*C*/*C*_i_ = 27.56% ± 3.9%) as soaking time continue to increase for noncontact detectivity. When the soaked sensor is dried, its performance retains back to the original state.

**Figure 3:**
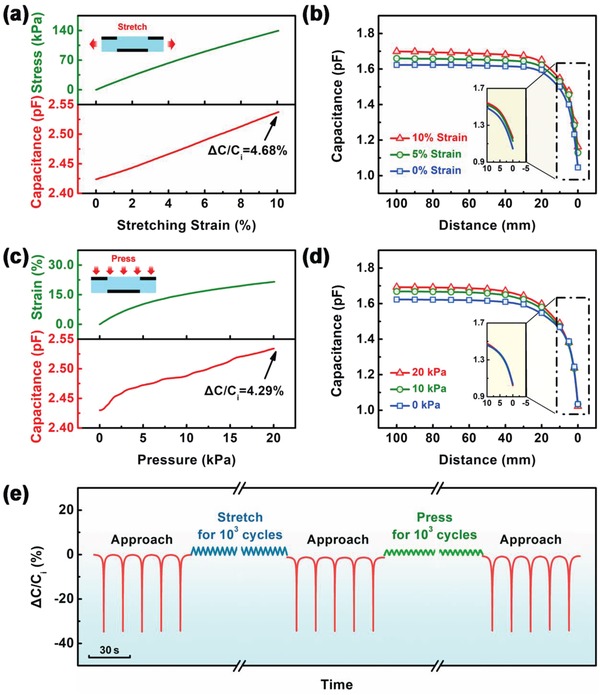
a) Stress–strain curve of stretching (upper), and capacitance as a function of stretching strain (below). b) Capacitance as a function of distance at stretching strain of 0%, 5%, and 10%. c) Strain–stress curve of pressure (upper), and capacitance as a function of pressure (below). d) Capacitance as a function of distance at pressures of 0, 10, and 25 kPa. e) Relative capacitive change rate during approach‐leave operations, repeated stretching cycle operations (10^3^ times), and repeated pressure cycle operations (10^3^ times).

Furthermore, the interference of deformation (stretching and pressure) on the performance of the sensor is investigated, because the deformation can significantly change the capacitance of the sensor according to the previously reported results.^[^ ^35^, ^36^, ^37^ ^]^ The prepared capacitive sensor is implanted in textile clothing for noncontact healthcare monitoring. Normally, the deformation of textile clothing is less than 10% stretching strain and can withstand 20 kPa pressure.^[^ ^38^ ^]^ The capacitance of the sensor implanted in textile cloth changes with stretching (0–10% strain) and pressure (0–20 kPa) as shown in **Figure** **3**. In Figure 3a, the initial capacitance of the sensor increases by 4.68% when stretched to 10% strain due to the decrease of vertical distance between the two electrodes. In comparison to capacitance change of common capacitive sensors, which consists of a pair of electrodes with the same shape, the capacitance of the sensor is about 10% when stretched to 10% strain, i.e., gauge factor (GF) of 1.^[^ ^33^, ^40^, ^48^ ^]^ The GF can be defined with the following Equation (1)

(1) $$\{\begin{array}{c} C=\frac{\varepsilon_{0}\varepsilon_{\text{r}}S}{d}=\frac{\varepsilon_{0}\varepsilon_{\text{r}}L\cdot W}{d}\\ \text{GF}=\frac{C\prime -C_{\text{i}}}{c_{i}e}=(C\prime \text{/}C_{0}-1)/e \end{array}\Rightarrow \text{GF}=\left[\frac{L(1+e)\cdot W(1-\upsilon e)}{d(1-\upsilon e)}\times \frac{d}{L\cdot W}-1\right]\text{/}e=1$$

Where *C* is the capacitance, ε_0_ is the permittivity of vacuum, ε_r_ is the relative permittivity of dielectric layer, *S*, *L*, *W*, and *d* are the overlap area, length, width, and distance between the electrodes, respectively, *C*_i_ and *C'* are the capacitance of sensor before and after stretching, respectively, *e* is the stretching strain, υ is the Poisson ratios. It shows that our capacitive sensor is less sensitive (GF = 0.47) to the stretching strain than the existing sensor because the deformation of the ring‐shaped top electrode changes the projected electric field of the sensor, which weakens the increase of capacitance induced by stretching strain. According to the simulation of COMSOL, the results are as expected that the initial capacitance only changes by 16.67% when stretched to 50% strain, i.e., the GF of 0.33 (Figure S8, Supporting Information). Therefore, when the sensor is stretched to a strain of 5% or 10% (Figure 3b), its noncontact detectivity is similar to the original state (Δ*C*/*C*_i_ = −31.77%). While the impact of pressure on the initial capacitance of our sensor is weaker as compared to stretching strain. It only changes by 4.29% when pressed to 20 kPa, as shown in Figure 3c. The pressure factor (PF) of our sensor is 0.0021 kPa^−1^, which is lower than that of other sensors based on porous dielectric materials (PF = 0.1_–_1 kPa^−1^).^[^ ^41^, ^42^, ^43^, ^44^, ^45^ ^]^ The formula of PF is similar to that of GF, i.e., the relative capacitance variation divided by the applied pressure. It is an interesting phenomenon to note that our sensor is insensitivity to pressure. In order to understand this phenomenon, the relative permittivity of porous PDMS under different pressures was investigated. The experimental result shows that the relative permittivity of porous PDMS decreases with the increase of pressure (Figure S9, Supporting Information). Under pressure of 25 kPa, the relative permittivity is 31.04%, which is lower than before. But the decrease of the relative permittivity of porous PDMS will weaken the pressure response of the sensor. One possible reason is that the pores of porous PDMS are closed cells (Figure S5, Supporting Information), which is an incompressible state. When the porous PDMS dielectric layer is pressed, the volume ratio of air increases, which leads to the decrease of relative permittivity, because total volume gets smaller and the air volume in the pores remains constant. Meanwhile, the relative capacitance change rate of the sensor under different pressure states is similar when an object approaches the sensor (Figure 3d). In that, it can be found that the relative capacitance change rate increases with the increase of pressure (Δ*C*/*C*_i_ = −31.02% @ 0 kPa, −38.03% @ 10 kPa, −39.61% @ 20 kPa). This characteristic feature can be attributed to the decrease of the relative permittivity of porous PDMS with the increase in pressure. In accordance with the previous result (Figure 2a), the noncontact detectivity of our sensor increases with the decrease of the relative permittivity of dielectric material. Meanwhile, the performance of the sensor is insensitive to bending. When an object approaches, the maximum capacitance change rates of the sensor are all between 37.46% and 38.81% (Figure S10, Supporting Information), at the bending radius of 6.0, 2.0, 0.7, and 0.2 cm. Figure 3e shows the noncontact detectivity of the sensor before and after repeated stretching (10% strain) and pressure (20 kPa) for 10^3^ times. When an object approaches the sensor, the relative capacitance change rate remains constant (Δ*C*/*C*_i_ = −34.49%) during the experiment. This means our capacitive sensor is suitable for implanting in clothes as a wearable healthcare sensor, which can detect the physiological signals of human in noncontact mode.

**Figure 4:**
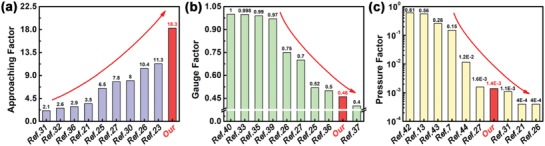
The comparison of performance between our sensor and with previously reported capacitive sensors in terms of a) approaching factor, b) gauge factor, and c) pressure factor.

As shown in **Figure** **4**, compared with previously reported capacitive sensors, our sensor shows better performance in terms of high noncontact detectivity and good noninterference capability of deformation. In the previously reported works, the initial object approaching distances are not uniform which leads to a difficulty in the comparison with the noncontact detectivity of these capacitive sensors. We define a new concept of approaching factor for capacitive sensor in order to compare with the previous reports, which means the relative capacitance change rate caused by an object approaching from 1 to 0 cm. For this, the approaching factor of our sensor is 18.3 (Figure 4a), which is about 2–5 times higher than that of others. At the noninterference capability of stretching and pressure, the GF and the PF of our capacitive sensor are smaller than the most of sensors, which implies the excellent performance of our capacitive sensor in comparison to previously reported (Figure 4b,c). Our sensors normally exhibit a small response to stretching and pressure, which means the influence of pressure on noncontact detection is small. The more detailed comparison of capacitive sensors is listed in **Table** **1**. The average values of approaching factor, gauge factor, and pressure factor are 7.073, 0.749, and 0.816, respectively, for the reported capacitive sensors. Obviously, the approaching factor of our sensor is 2.587 times higher than that of others, which shows the sensor is more sensitive to detect an object approaching. The gauge factor and pressure factor of our sensor is 0.614, and 0.002 times lower than that of others, respectively. This means the effect of deformation is little on our sensor.

**Table 1:** The more detailed comparison of capacitive sensors

Ref.	Approaching factor	Gauge factor	Pressure factor
Our	18.3	0.46	0.0014
^[^ ^21^ ^]^	3.5	–	0.0004
^[^ ^25^ ^]^	6.5	0.52	0.0052
^[^ ^26^ ^]^	10.4	0.75	0.0004
^[^ ^27^ ^]^	7.8	0.7	0.0016
^[^ ^30^ ^]^	8.0	–	–
^[^ ^31^ ^]^	2.1	–	0.0011
^[^ ^32^ ^]^	2.6	–	–
^[^ ^46^ ^]^	14.9	–	0.0224
^[^ ^47^ ^]^	7.8	–	–
^[^ ^42^ ^]^	2.9	0.5	–
^[^ ^23^ ^]^	11.3	–	–
^[^ ^40^ ^]^	–	1	–
^[^ ^33^ ^]^	–	0.998	–
^[^ ^35^ ^]^	–	0.99	–
^[^ ^39^ ^]^	–	0.97	–
^[^ ^37^ ^]^	–	0.4	–
^[^ ^42^ ^]^	–	–	0.61
^[^ ^13^ ^]^	–	–	0.56
^[^ ^43^ ^]^	–	–	0.26
^[^ ^7^ ^]^	–	–	0.15
^[^ ^44^ ^]^	–	–	0.012
^[^ ^48^ ^]^	–	–	0.0925
^[^ ^49^ ^]^	–	–	0.815
^[^ ^50^ ^]^	–	–	0.07
^[^ ^51^ ^]^	–	0.998	–
^[^ ^52^ ^]^	–	0.58	–
^[^ ^53^ ^]^	–	0.83	–
^[^ ^54^ ^]^	–	–	0.76
^[^ ^55^ ^]^	–	0.5	–
^[^ ^56^ ^]^	–	–	9.9
^[^ ^42^ ^]^	–	–	0.610

Marking “–” means that there is no relevant research data in this paper.

**Figure 5:**
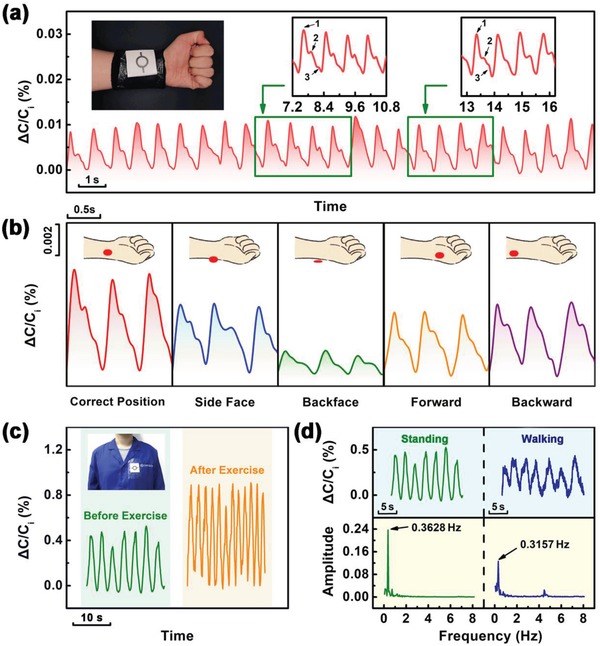
a) Noncontact monitoring of pulse signals. The first inset is a smart sports wristband implanted with our sensor on its surface. The second inset is the enlarged image of the relative capacitance change rate from 8.2 to 10.6 s. The third inset is the enlarged image of the relative capacitance change rate from 12.8 to 16.2 s. b) Noncontact monitoring of pulse signals at different positions. c) Noncontact monitoring of respiratory signals before and after exercise. Inset shows the smart clothes implanted with our sensor on its surface. d) Noncontact monitoring of respiratory signals at different state (upper) and amplitude‐frequency curves of signals by Fourier transform (below).

Therefore, the prepared capacitive sensor can be successfully used as a wearable sensor for noncontact healthcare monitoring of physiological signals, such as respiratory signals and pulse signals, as shown in **Figure** **5**. In Figure 5a, a person wears a smart sports wristband attached with our sensor on its surface. The wristband is made up of soft absorbent cotton with a thickness of 2 cm. From Figure 5a, it can be found that the pulse signals of human are recorded clearly by our sensor, which includes peak 1 and peak 2. The pulse rate is about 72 times per minute. In practical application, different operators and movements can make the sensor to diverge from the correct detection position, which causes the detection errors. The analyzed results of pulse detection, when the sensor is placed around the radial artery, including side face, back face, forward position, and backward position, are shown in Figure 5b. From that, the decay of signal strength can be observed at different positions, especially at the back face position, due to the increase of detection distance. But the pulse rate can still be detected that shows the advantages of noncontact detection sensors in terms of fault tolerance, which helps to reduce the difficulty of using our sensor. In Figure 5c, a person wears smart clothes in which the sensor is attached on the surface of clothes. The cloth is very loose and comfortable. The recorded respiratory signals are clearer from the sensor attached to the surface of the cloth. Before and after exercise, the respiratory rates are 21 times per minute and 36 times per minute, respectively. Moreover, the sensor can record the respiratory signals when people are in the walking state (Figure 5d). When a person is walking, the recorded signal fluctuates slightly up and down, which may be caused by the natural swing of clothing as people move around. By Fourier transform, it can be found that the main characteristic frequencies of recorded signals are 0.3628 and 0.3157 Hz, respectively, when a person is standing and walking. These frequencies are equivalent to the respiratory rate of an adult (18–22 times per minute). When a person is walking, there is a small characteristic amplitude change at frequency 4.46 Hz from the amplitude‐frequency curve of the signal. This characteristic change may be due to the frequency of natural swing of clothing as people move around. This proves that the smart clothing based on our sensor can monitor the physiological signals of human in real time in the noncontact mode, whether the human body moves or not. Furthermore, the sensor is attached to the back of a chair in order to develop intelligent furniture (Figure S11, Supporting Information). It can be found that the respiratory signals were recorded when the person sits on the chair before and after exercise. After exercise, the respiratory intensity and respiratory rate of a person have increased significantly. All these abilities of our sensor without changing the sensitivity are mainly due to the ring‐shaped top electrode and choosing the porous dielectric material with low relative permittivity in the sensor. In addition, we also think that our capacitive sensor can be used in soft robots. With the excellent noncontact detectivity of our sensor, soft robots can easily avoid predators like the obstacles by using our sensor. We have demonstrated such a feature of avoiding obstacles on a toy car in noncontact mode, as shown in Video S1 of the Supporting Information.

## 3 Conclusion

In summary, we reported a wearable healthcare capacitive sensor, which consists of a ring‐shaped top LMs@PDMS electrode, a disk‐shape bottom LMs@PDMS electrode, and a porous PDMS dielectric layer with low permittivity. The electrodes with different shapes are prepared by stencil printing technology, and porous PDMS is obtained by heating the composite of water@PDMS. This design significantly improved the noncontact detectivity of the capacitive sensor as observed from the experimental tests, which occur in accordance with simulation by COMSOL. When the object approaches the sensor from a distance of 200 mm, the capacitance change rate of sensor with optimal parameters (40 vol% mixing ratio of water, 3 mm thickness of sensor, 5 mm horizontal offset of two electrodes) is 2–5 times higher than that of previously reported capacitive sensors (Δ*C*/*C*_i_ = −38.7%). Importantly, our capacitance sensor is insensitive to the stretching strain and pressure (Δ*C*/*C*_i_ < 5% @ 10% strain or 20 kPa), which makes it highly suitable for wearable sensors that can be attached with the textile clothing. Its high noncontact detectivity and good noninterference capability of deformation promise its superior performance for noncontact healthcare monitoring. Human physiological signals, such as respiratory signals and pulse signals are successfully recorded in the noncontact situation when the person sits on intelligent furniture or wore smart clothes, which are implanted with our capacitive sensor. This means the noncontact healthcare monitoring system based on our wearable capacitive sensor is expected to revolutionize the detecting method of existing wearable sensor and an appropriate method for monitoring special patients, such as infants or persons with burn injuries. In addition, we believe this work can be extended for motion planning of soft robots to avoid predators like living animals.

## 4 Experimental Section

##### Preparation of Wearable Capacitive Sensor for Noncontact Healthcare Monitoring

A ring‐shaped mask and a disk‐shaped mask were engraved on the polyethylene terephthalate (PET) film using a laser engraving machine (NEJE Master, Shenzhen Baoliwang Trading Co. LTD). The PET film with a thickness of 140 nm was attached to a glass plate during the engraving process. The ring‐shaped electrode mask has an inner diameter of 2.5 cm and an outer diameter of 3 cm. The disk‐shaped bottom electrode mask has a diameter of 2 cm. First, uncured LMs@PDMS conductor ink (LMs/PDMS = 15:1 by mass) was brushed directly onto the PET mask film, which was pasted on the glass plate. The LMs@PDMS was prepared in the same way as previously reported.^33^, ^34^ ^]^ Then, the PET film was peeled off, and LMs@PDMS ink was cured at 80 °C for 2 h to obtain LMs@PDMS electrode with a specific shape (a ring‐shaped top electrode and a disk‐shaped bottom electrode). Next, a hollow rectangular teflon mold was placed on the glass plate with LMs@PDMS electrodes. Meanwhile, the uncured water@PDMS mixture of a certain mass was poured into the teflon mold. The uncured water@PDMS mixture was obtained by mechanical stirring of water and PDMS (Sylgard 184, Dow Corning Corporation) at a mass ratio of 6:4 for 30 min. It was noted that the stirred mixture should be evacuated for 30 min in order to remove the air bubbles in the mixture. Afterward, the whole setup including the glass plate, teflon mold, and uncured water@PDMS mixture, was heated at 70 °C for 30 min and at 120 °C for 2 h to prepare a porous PDMS dielectric layer. Finally, the upper and lower half parts were joined together by PDMS to obtain a wearable capacitive sensor for noncontact healthcare monitoring.

##### Characterization of the Microstructure

The cross‐sectional microstructures of samples were characterized by the field‐emission scanning electron microscopy (Sirion 200, FEI).

##### Rheology Measurements

The rheological properties of uncured LMs@PDMS conductive ink and uncured water@PDMS mixtures were characterized by rotational rheometer (Physica MCR‐301, Anton Paar) at room temperature. During the measurements, the storage modulus, loss modulus, and apparent viscosity are recorded simultaneously, when oscillatory measurements were carried out at a frequency of 1 Hz within the shear strain of 0.1–100%.

##### Measurements of Electrical Properties and Mechanical Properties

The capacitance change of sensor was measured by an Inductance Capacitance Resistance Meter (IM 3570, HIOKI Impedance Analyzer) with an AC signal amplitude oscillating at a frequency of 500 kHz. The stretching strain and pressing strain were applied by a universal material testing machine (Instron 5943).

##### Measurements of Relative Permittivity at Different States

Initially, several square shaped LMs@PDMS elastic electrodes were prepared with size of 1 cm × 1 cm, and porous PDMS dielectric layers with different number of pores were prepared with size of 2 cm × 2 cm. Then, two electrodes and one porous PDMS dielectric layer were joined together by PDMS to obtain a conventional capacitive sensor (square electrode/dielectric layer/square electrode). Next, the capacitance values and the thickness of sensors were measured by the inductor‐capacitance‐resistance meter and thickness testing machine, respectively. Finally, the relative permittivities of various porous PDMS were calculated according to Equation (1). When pressed, the measurement of relative permittivity change of porous PDMS is similar to the above content. Its conversion equations for testing values are shown in the inset of Figure S9 of the Supporting Information.

##### Theoretical Simulations of the Capacitive Sensor

The COMSOL software package was used to analyze the measuring process of the sensor. The LMs@PDMS elastic electrodes and the approaching object were considered as a conductor, which were applied at a certain electric potential of their boundary. The electric potential of the upper part of the sensor was 1 V. While the electric potential of the lower part of the sensor and the approaching object were 0 V. The relative permittivity of the air around the sensor was 1. The relative permittivities of the dielectric layer were 3 and 2, respectively. The sensor was put in the center of an infinite space and the electric potential at the infinite boundary is 0. The same simulation setup for the stretched sensor without the approaching object was used. The Neo‐Hookean model was used to describe the mechanical response of the PDMS with a shear modulus equal to 1.

## Conflict of Interest

The authors declare no conflict of interest.

## Supporting information

Supporting Information

Supplemental Video 1
