# Supplementary material for: A Wearable Capacitive Sensor Based on Ring/Disk‐Shaped Electrode and Porous Dielectric for Noncontact Healthcare Monitoring
Source: Glob Chall. 2020 Mar 18;4(5):1900079. doi: 10.1002/gch2.201900079 (PMC7175022; doi:10.1002/gch2.201900079)
Supplement: Supplementary file 1 — Supporting Information [file GCH2-4-1900079-s001.pdf]

Copyright WILEY-VCH Verlag GmbH & Co. KGaA, 69469 Weinheim, Germany, 2020.

Global Challenges
Open Access

Supporting Information

for *Global Challenges,* DOI: 10.1002/gch2.201900079

A Wearable Capacitive Sensor Based on Ring/Disk-Shaped Electrode and Porous Dielectric for Noncontact Healthcare Monitoring

Ya-Nan Zheng, Zhe Yu, Guoyong Mao, Yunyao Li, Dhanapal Pravarthana, Waqas Asghar, Yiwei Liu, Shaoxing Qu, Jie Shang, and Run-Wei Li**

Supporting Information

Copyright WILEY-VCH Verlag GmbH & Co. KGaA, 69469 Weinheim, Germany, 2020

A Wearable Capacitive Sensor Based on Ring/Disc-shaped Electrode and Porous Dielectric for Non-contact Healthcare Monitoring

Ya-Nan Zheng,† Zhe Yu,† Guoyong Mao, Yunyao Li, Dhanapal Pravarthana, Waqas Asghar, Yiwei Liu, Shaoxing Qu, Jie Shang, and Run-Wei Li**

†These authors contribute equally to this work.

Y.-N. Zheng, Z. Yu, Y. Y. Li, Dr. D. Pravarthana, W. Asghar, Prof. Y. W. Liu, Prof. J. Shang, Prof. R.-W. Li
CAS Key Laboratory of Magnetic Materials and Devices,
Ningbo Institute of Materials Technology and Engineering, Chinese Academy of Sciences, Ningbo 315201, P. R. China
E-mail: shangjie@nimte.ac.cn (Prof. J. Shang); runweili@nimte.ac.cn (Prof. R.-W. Li)

Y.-N. Zheng, Z. Yu
College of Materials Science and Opto-Electronic Technology,
University of Chinese Academy of Sciences, Beijing 100049, P. R. China

Y.-N. Zheng, Z. Yu, Y. Y. Li, Dr. D. Pravarthana, W. Asghar, Prof. Y. W. Liu, Prof. J. Shang, Prof. R.-W. Li
Zhejiang Province Key Laboratory of Magnetic Materials and Application Technology,
Ningbo Institute of Materials Technology and Engineering, Chinese Academy of Sciences, Ningbo 315201, P. R. China

Dr. G. Mao, Prof. S. Qu
State Key Laboratory of Fluid Power and Mechatronic System,
Key Laboratory of Soft Machines and Smart Devices of Zhejiang Province,
Department of Engineering Mechanics, Zhejiang University, Hangzhou 310027, China

Y. Y. Li
College of Information Engineering,
Nanjing University of Finance and Economics, Nanjing, 210046, China

Keywords: wearable capacitive sensor, non-contact healthcare monitoring, capacitively coupled effect, electrode shape, porous dielectric

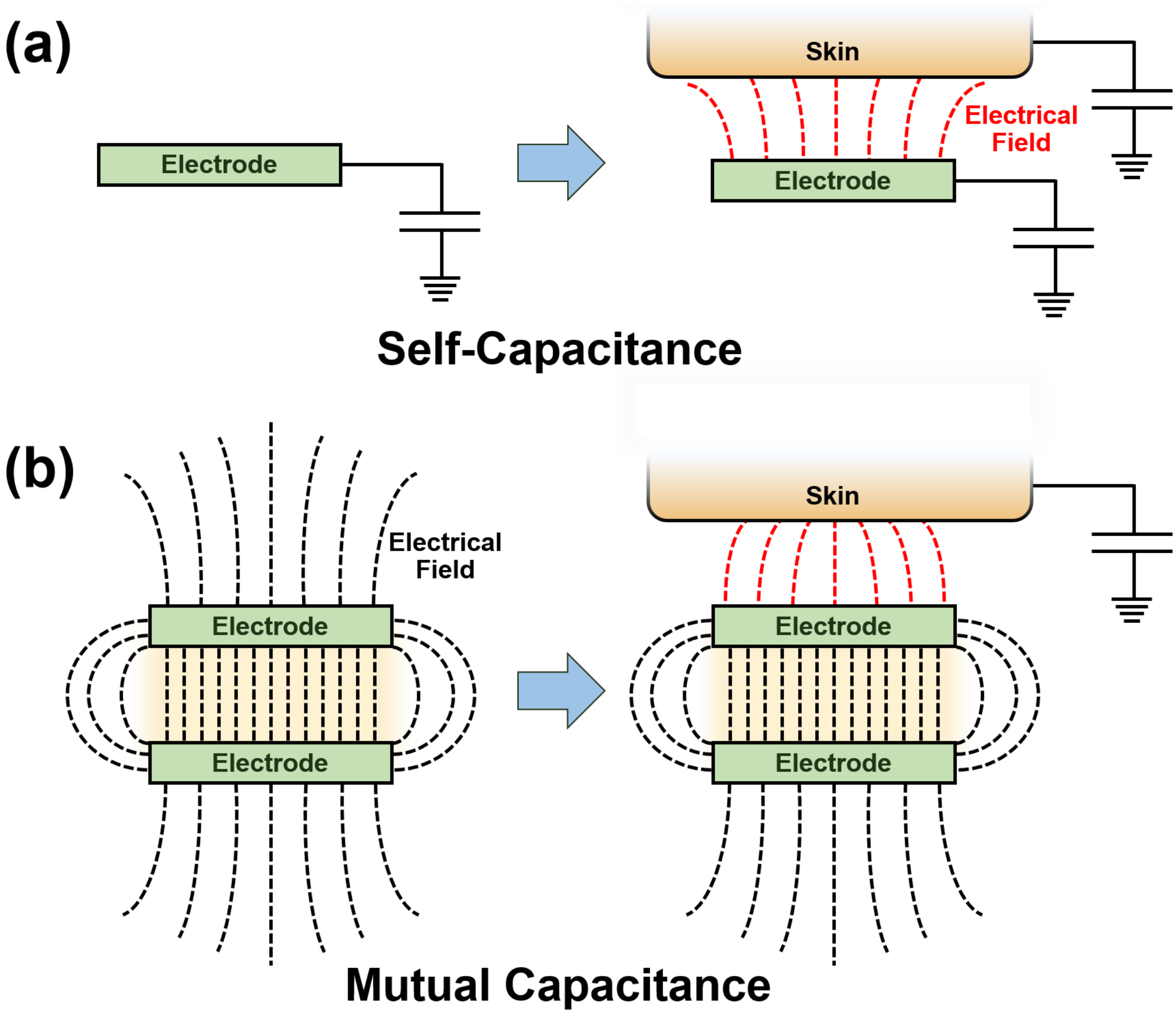

Figure S1. Schematic illustration of the working principle of two basic types of capacitively coupled sensing ways: **(a)** self-capacitance; **(b)** mutual capacitance.

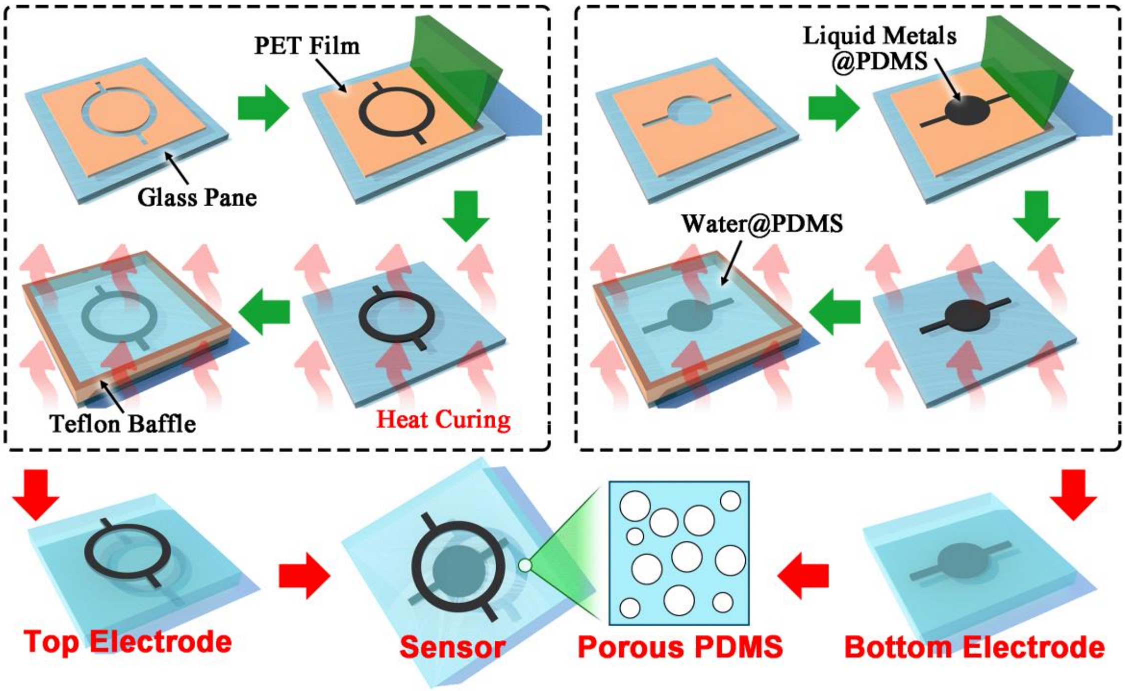

Figure S2. Schematic illustration of the detailed fabrication procedure for preparing wearable capacitive sensors.

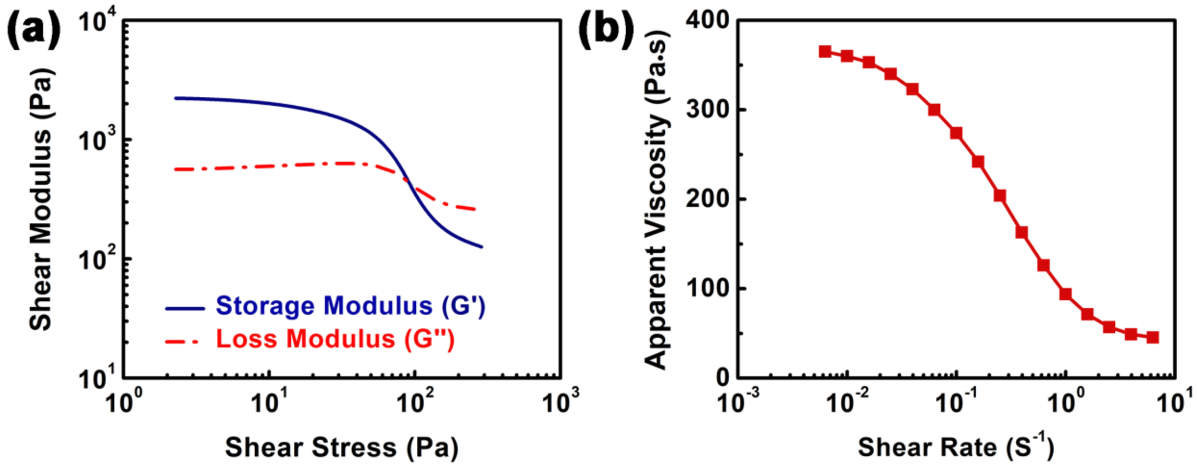

Figure S3. (a) Shear modulus as a function of shear stress for uncured LMs@PDMS conductive ink. **(b)** Apparent viscosity as a function of shear rate for uncured LMs@PDMS conductive ink.

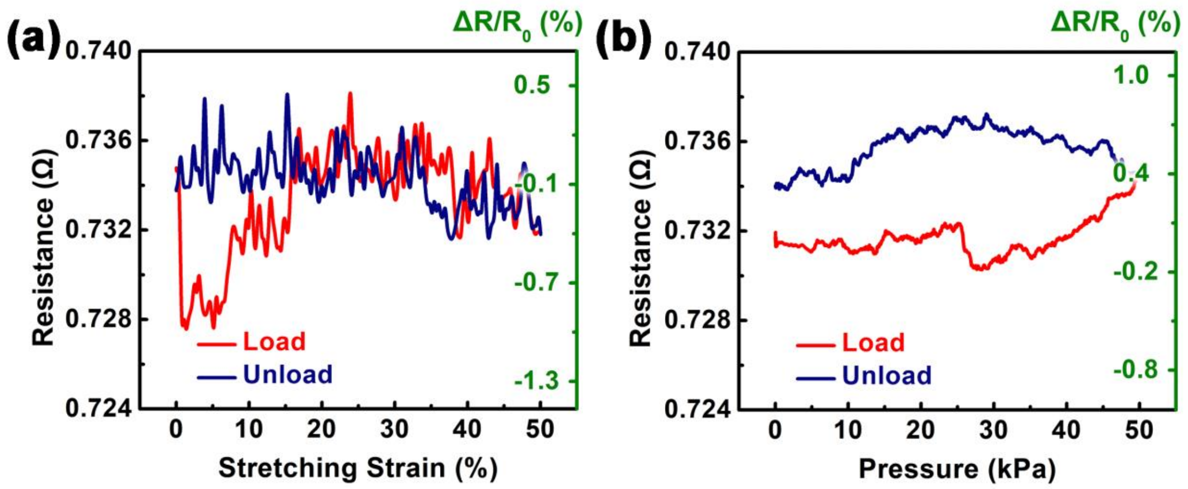

Figure S4. (a) Resistance as a function of stretching strain for LMs@PDMS elastic conductor. **(b)** Resistance as a function of pressure for LMs@PDMS elastic conductor.

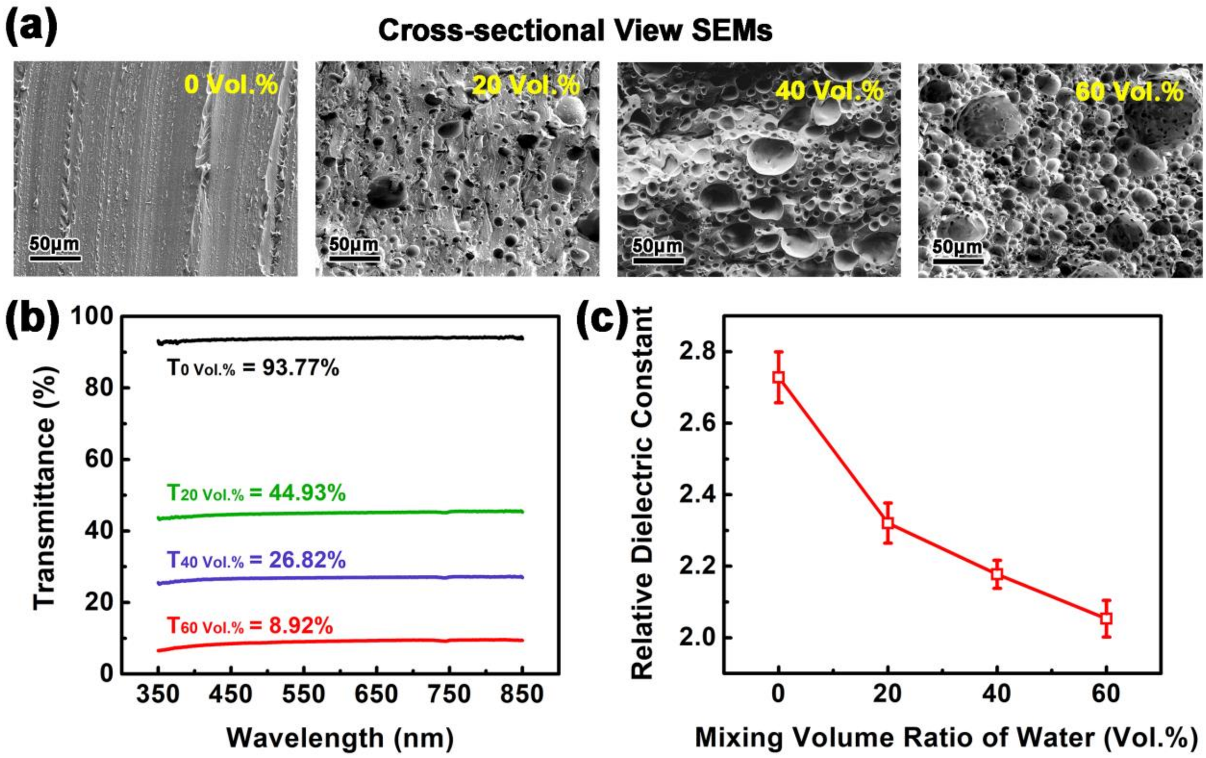

Figure S5. (a) Cross-sectional view SEM images of PDMS dielectric materials with different numbers of pores. **(b)** Transmittance as a function of wavelength for cured water@PDMS dielectric materials with different mixing water volume ratios. **(c)** Relative permittivity as a function of water volume ratio for cured water@PDMS dielectric materials.

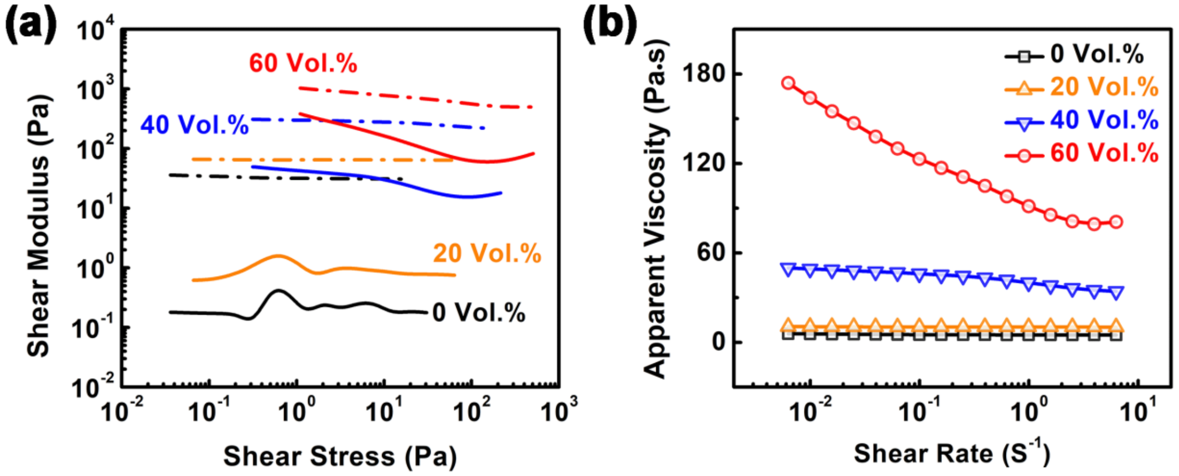

Figure S6. (a) Shear modulus as a function of shear stress for uncured water@PDMS mixture at different mixing volume ratios of water. The solid and dashed lines are storage modulus (G') and loss modulus (G"), respectively. **(b)** Apparent viscosity as a function of shear rate for uncured water@PDMS mixture at different mixing volume ratios of water.

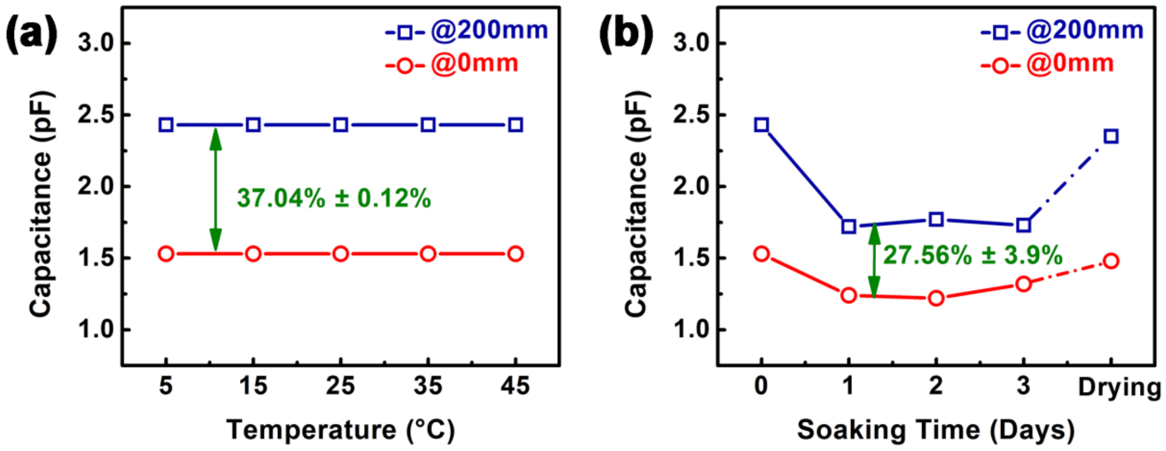

Figure S7. (a) Capacitance as a function of temperature at distances of 200 mm and 0 mm. **(b)** Capacitance as a function of soaking time at distances of 200 mm and 0 mm.

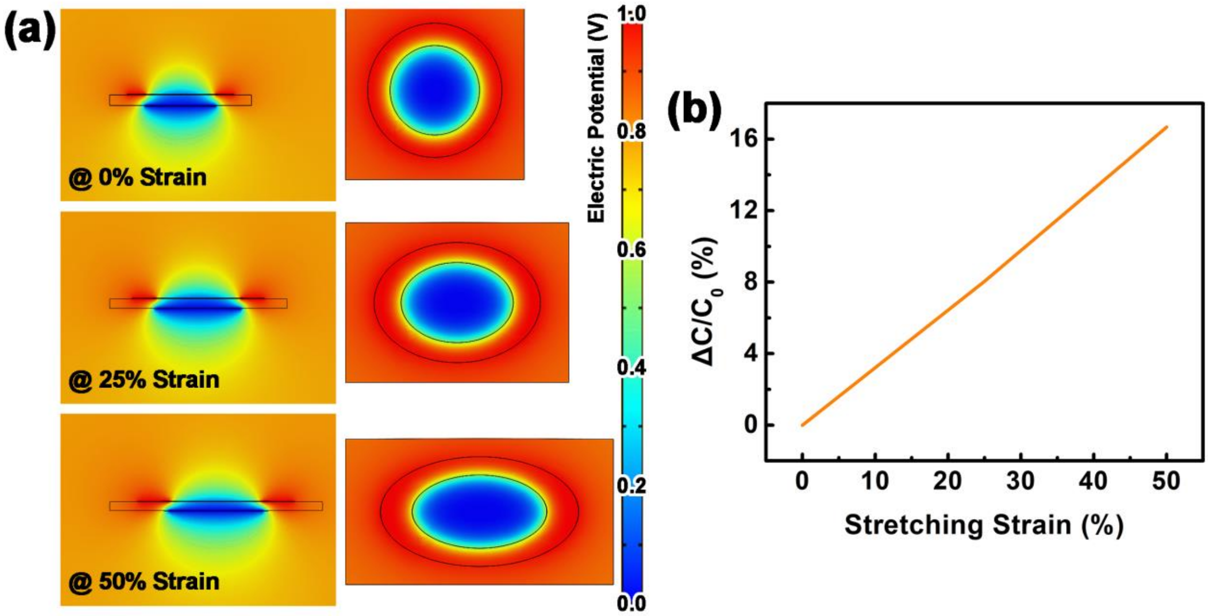

Figure S8. Simulated results of the prepared capacitive sensor during stretching. **(a)** Distribution images of the fringing electric field at different stretching strains. **(b)** Relative capacitance change rate as a function of stretching strain.

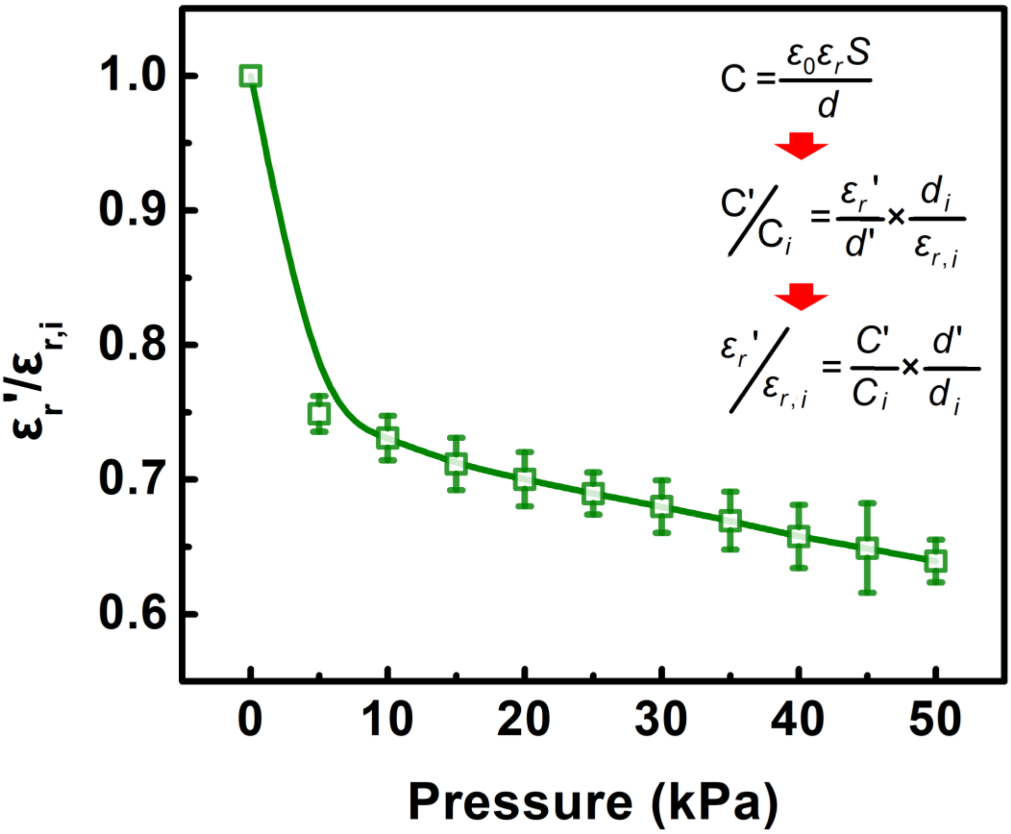

Figure S9. Relative permittivity change rate as a function of pressure. The inset shows the conversion equations for measured values.

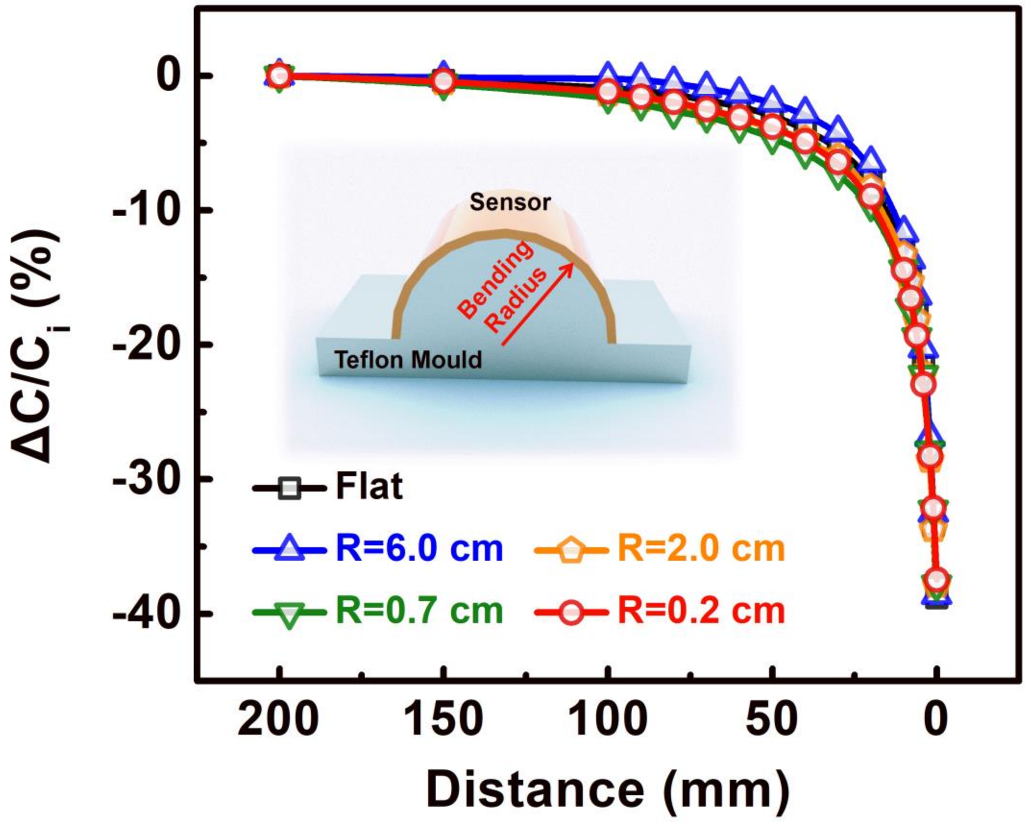

Figure S10. Capacitance as a function of distance at bending radiuses of 6.0 cm, 2.0 cm, 0.7 cm, and 0.2 cm.

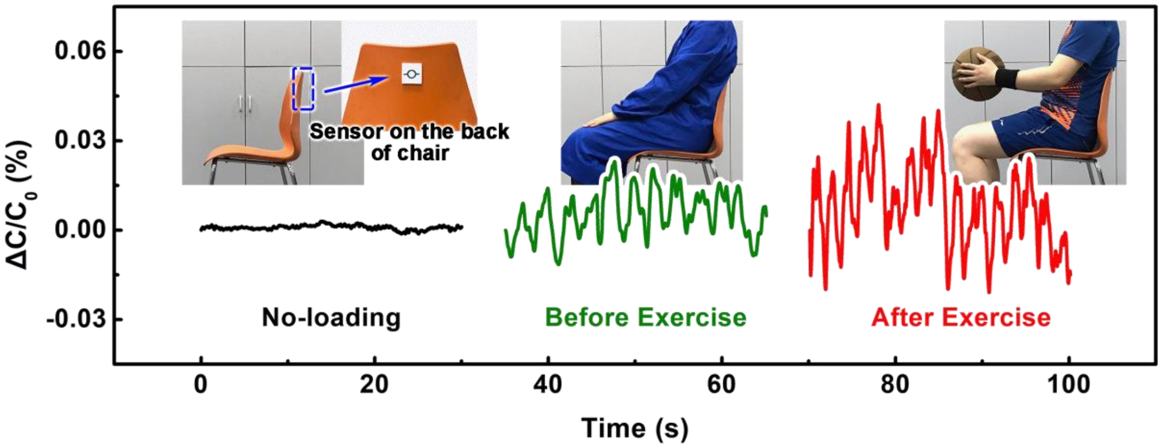

Figure S11. Non-contact monitoring of respiratory signals in different states. The first inset is an intelligent chair implanted with our sensor on its back. The second and third insets show the testing picture before exercise and after exercise, respectively.
